# Vertebral osteomyelitis and epidural abscess caused by gas gangrene presenting with complete paraplegia: a case report

**DOI:** 10.1186/s13256-015-0567-y

**Published:** 2015-04-11

**Authors:** Manabu Akagawa, Takashi Kobayashi, Naohisa Miyakoshi, Eiji Abe, Toshiki Abe, Kazuma Kikuchi, Yoichi Shimada

**Affiliations:** 1 Department of Orthopedic Surgery, Akita Kousei Medical Center, 1-1-1 Iijima-Nishifukuro, Akita, 011-0948 Japan; 2 Department of Orthopedic Surgery, Akita University Graduate School of Medicine, 1-1-1 Hondo, Akita, 010-8543 Japan

**Keywords:** *Clostridium perfringens*, Debridement, Epidural abscess, Fusion surgery, Gas gangrene, Vertebral osteomyelitis

## Abstract

**Introduction:**

Gas gangrene is most often caused by *Clostridium perfringens* infection. Gas gangrene is a medical emergency that develops suddenly. The mortality rate is higher with trunk involvement than with involvement of the extremities, which carries a better prognosis. With respect to vertebral involvement, there are few reports in the literature. The purpose of this paper is to report a very rare case of vertebral osteomyelitis caused by gas gangrene.

**Case presentation:**

A 78-year-old Japanese woman with diabetes mellitus was admitted to our hospital with the chief complaints of back pain, dysuria, and complete paralysis of both legs. A computed tomography scan showed soft tissue swelling anterolaterally at intervertebral disc level T11/12 and a gas-containing epidural abscess that compressed her spinal cord. Cultures later grew *Clostridium perfringens* and *Escherichia coli*. Hemilaminectomy was done from T10 to T12, and an epidural abscess was removed. She went on to have fusion surgery 6 weeks after the initial operation and subsequently experienced complete pain relief. She was discharged 2 months later, at which time she was able to walk with a cane. Examination 18 months after surgery showed normal gait without a cane.

**Conclusions:**

Discitis caused by gas gangrene infection was successfully treated by immediate debridement and subsequent fusion surgery.

## Introduction

Spontaneous gas gangrene is most often caused by a bacterium called *Clostridium perfringens* [1]. Gas gangrene is one of the most fulminant infectious diseases; it can cause myonecrosis, gas production, and sepsis. Progression of toxemia and shock is often very rapid. The mortality rate is higher with trunk involvement than with involvement of the extremities, which carries a better prognosis [2]. Antibiotics alone are not effective because they do not penetrate ischemic muscles sufficiently [3]. With respect to vertebral involvement, there are few reports in the literature [4-10]. The purpose of this paper is to report a very rare case of vertebral osteomyelitis caused by gas gangrene.

## Case presentation

A 78-year-old Japanese woman with diabetes mellitus was admitted to our institution with a 1-week history of back pain and a 1-day history of paralysis of both legs. She had been bedridden for a week prior to admission in another hospital. There was no history of trauma. No gastrointestinal symptoms were present. Her temperature was 36.8°C, blood pressure was 100/70mmHg, and pulse was 103 beats per minute. On physical examination, her patellar tendon reflexes were present, but Achilles tendon reflexes were absent. All sensory modalities were absent below the inguinal level. Her leg muscles were completely paralyzed bilaterally. There was no anal wink. Anal tone was diminished, and there was no voluntary contraction of the external sphincter.

**Figure 1:**
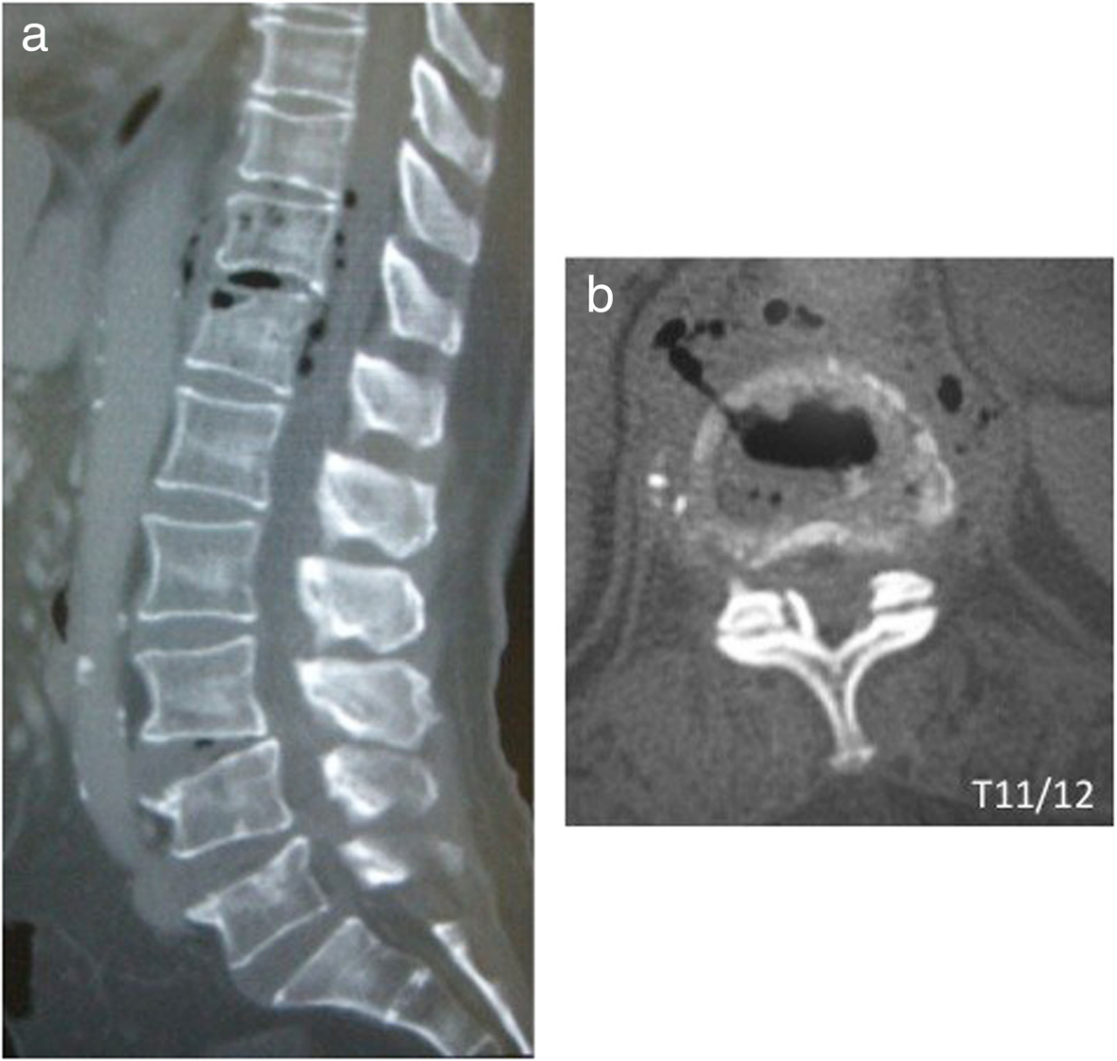
Computed tomography sagittal and axial images on initial admission. (**a**) Soft tissue swelling anterolaterally at intervertebral disc level T11/12 (**b**) and a gas-containing epidural abscess are seen.

**Figure 2:**
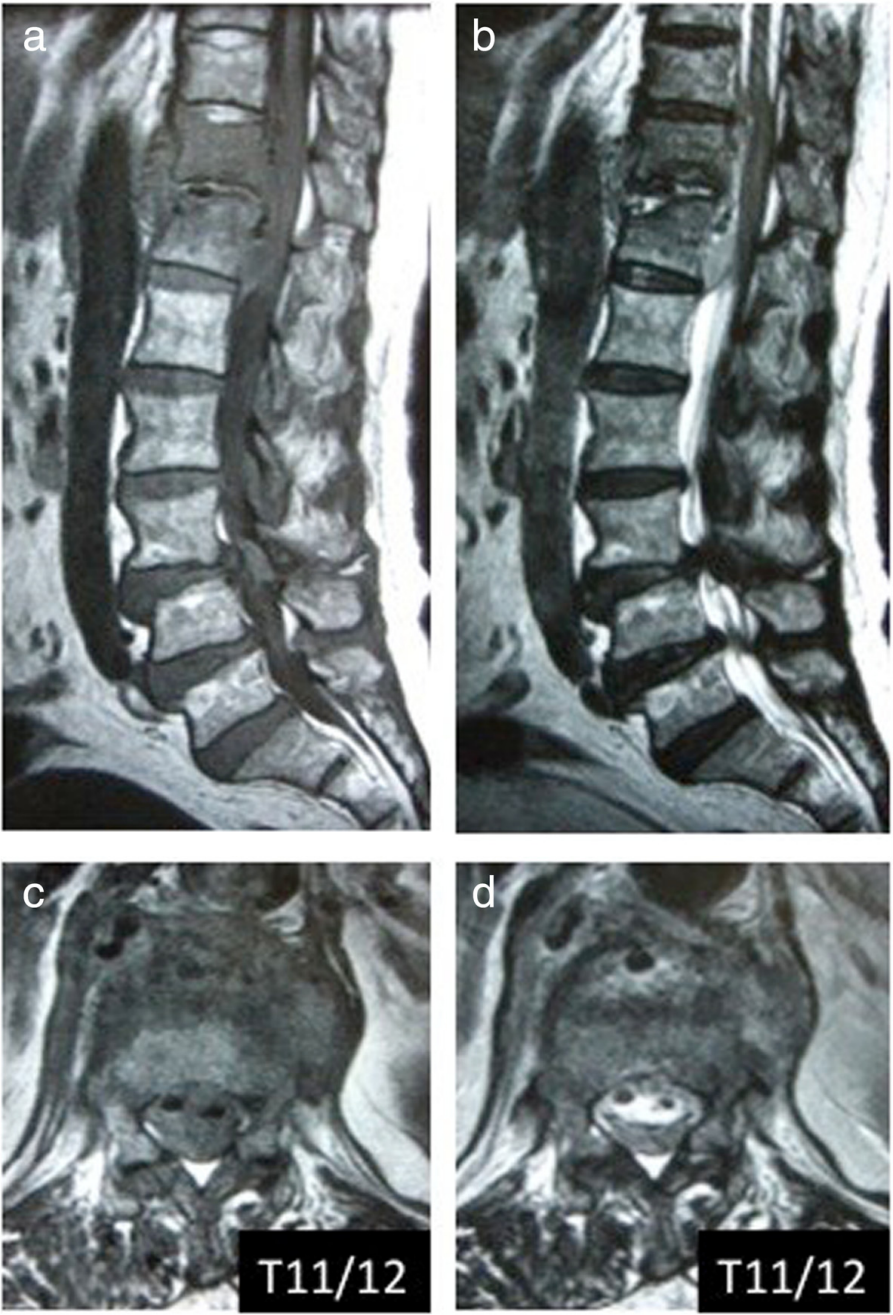
Magnetic resonance imaging sagittal and axial images on initial admission. Involvement of the T11/12 disc space and adjacent vertebral body with decreased signal intensity on the T1-weighted images (**a** and **c**) and increased signal intensity on the T2-weighted images (**b** and **d**) with a gas-containing epidural abscess are seen.

Admission laboratory values included a white blood cell count (WBC) of 18,800 per mm^3^, her hemoglobin was 9.4g/dL, and her hematocrit was 30%. Her Westergren erythrocyte sedimentation rate was 80mm/hour, and C-reactive protein (CRP) was 30.02mg/dL. Initial radiographic examination of her lumbar spine at admission showed only spondylotic change. A computed tomography (CT) scan showed soft tissue swelling anterolaterally at intervertebral disc level T11/12 and a gas-containing epidural abscess that compressed her spinal cord (Figure 1). Magnetic resonance imaging showed involvement of the T11/12 disc space and adjacent vertebral body with decreased signal intensity on T1-weighted images and increased signal intensity on T2-weighted images with a gas-containing epidural abscess (Figure 2).

The patient was urgently admitted to our hospital and taken to the operating room for decompression and disc debridement. Hemilaminectomy was done from T10 to T12, and the epidural abscess was removed, followed by T11/12 disc debridement. The epidural abscess was yellow and turbid. Cultures later grew *C. perfringens* and *Escherichia coli*. The wound was closed over subfascial drains.

On the second postoperative day, she was confused, and hydrothorax appeared. Thoracic drainage was then performed by a surgeon. Intravenous antibiotic treatment was begun with imipenem/cilastatin at 0.5g every 8 hours for 2 weeks, followed by piperacillin/tazobactam at 1.0g every 8 hours for 2 weeks, and then ampicillin-sulbactam at 1.0g every 8 hours for 2 weeks.

**Figure 3:**
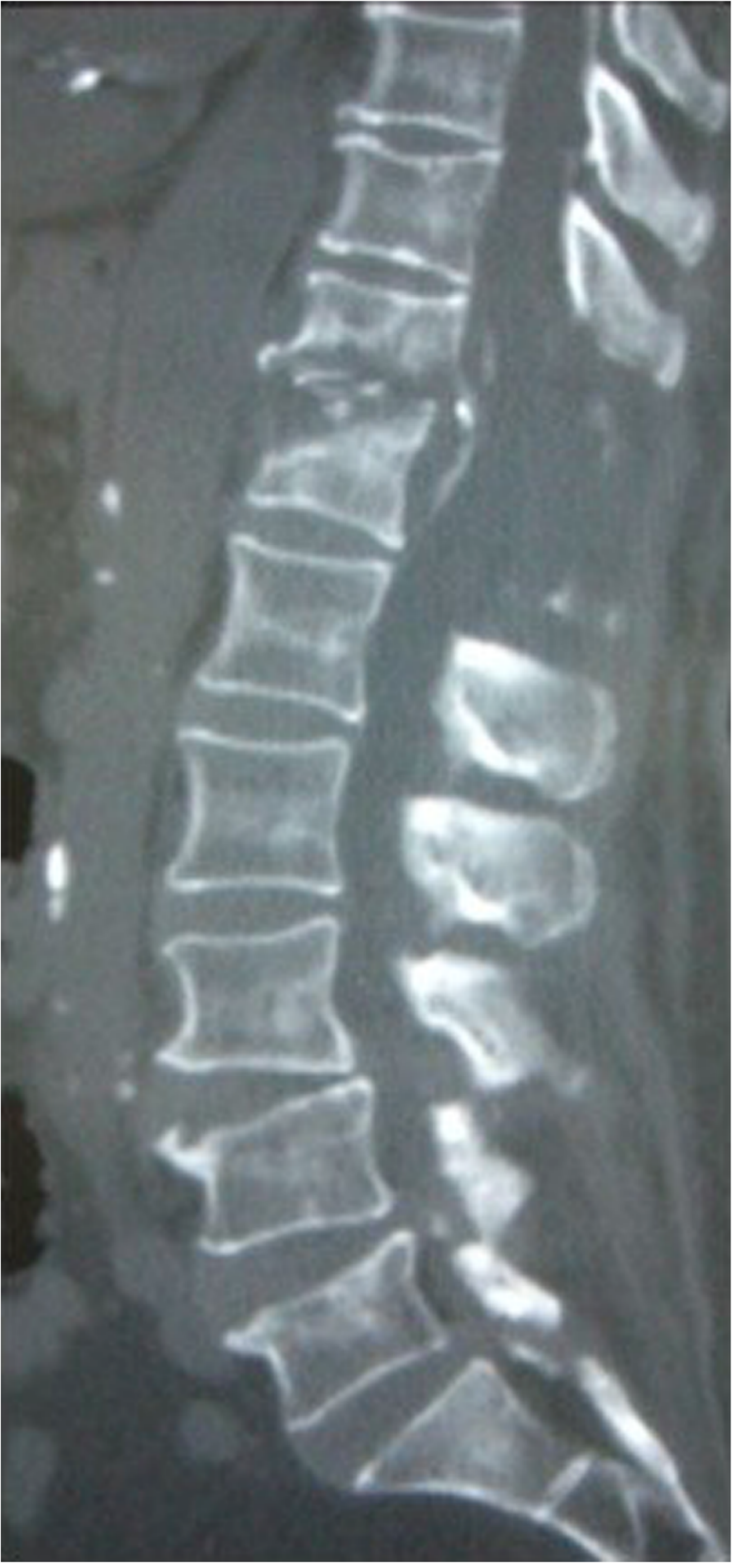
Computed tomography at 6 weeks after operation. T12 vertebral bone destruction is seen.

**Figure 4:**
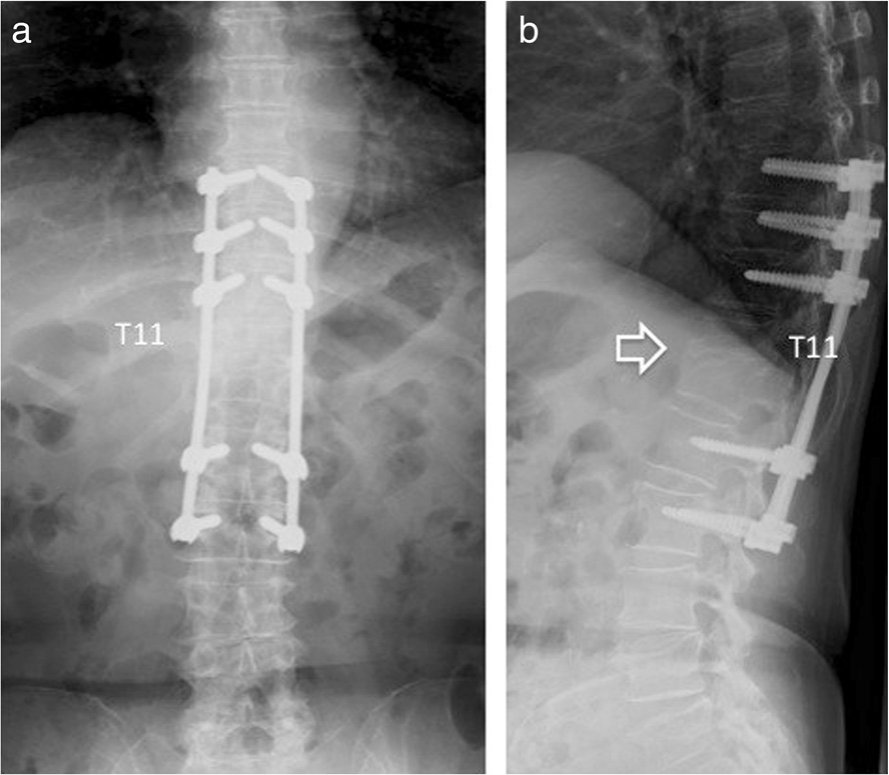
Plain radiograph at 18 months after operation. Complete union between the T11 and T12 vertebral bodies is seen. Open arrow shows bone bridge between T11 and T12 vertebra. (**a**) Anteroposterior radiograph, (**b**) Lateral radiograph.

Postoperatively, she experienced some sensory and motor return in her legs, but back pain and left chest pain in the sitting position continued. A CT scan 6 weeks after the operation showed T12 vertebral bone destruction (Figure 3); her WBC count and CRP were 8800 per mm^3^ and 0.26mg/dL, respectively. She went on to have fusion surgery with instrumentation and subsequently experienced complete relief of her pain. She was discharged 2 months later, at which time she was able to walk with a cane. Examination 18 months after surgery showed normal gait without a cane. Plain radiograph at 18 months after operation showed complete union between the T11 and T12 vertebral bodies (Figure 4).

## Discussion

Gas gangrene is divided into the following three types: posttraumatic, postoperative, and spontaneous [11]. A literature review reported that the spontaneous type accounts for 16% of cases [11]. Spontaneous clostridial myonecrosis is caused by *C. perfringens* and *Clostridium septicum* in 60% and 30% of cases, respectively [12,13]. Clostridial species are commonly found in soil, dust, water, and the intestines of humans and various animals [2,14,15]. Without treatment, there is a 100% mortality rate within 24 hours of onset of systemic symptoms [14]. If properly treated, the overall mortality rate is 20 to 30% [2,3,16]. The mortality rate is higher with trunk involvement (50%) than with involvement of the extremities (24%), which carries a better prognosis [2].

With respect to vertebral involvement, favorable outcomes after discitis caused by *C. perfringens* are obtained if the infection occurs only within the disc [5,6,8,10]. However, with surgical site infection (SSI) after lumbar spine surgery, one patient died, and one needed multiple operations [4,7]. The differences in outcomes may be related to the amount of ischemic tissue. Although the disc itself has a small amount of ischemic tissue and a favorable outcome, the amount of ischemic tissue with SSI is larger and is associated with a terrible outcome. The present case with discitis and an epidural abscess survived because the amount of ischemic tissue was small, and emergency debridement and antibiotic therapy were effective.

Fusion surgery is needed if instability remains [17]. Stabilization with instrumentation is a safe and effective treatment for pyogenic osteomyelitis [18-21]. In this case, even after her CRP level decreased, the patient could not sit because of her left lateral chest pain. On CT, vertebral bone destruction had appeared, and instability was considered the main reason for her lateral chest pain. We managed this patient with posterior fixation with instrumentation. She was treated successfully, and she was ambulatory and showed complete bone union on X-ray at 18-month follow-up.

## Conclusions

A very rare case of vertebral osteomyelitis caused by gas gangrene that was successfully treated by immediate debridement and subsequent fusion surgery combined with antibiotics was described.

## Consent

Written informed consent was obtained from the patient for publication of this case report and accompanying images. A copy of the written consent is available for review by the Editor-in-Chief of this journal.

## Abbreviations

CRP: C-reactive protein
CT: computed tomography
SSI: surgical site infection
WBC: white blood cell count
